# Technological Remedies for Social Problems: Defining and Demarcating Techno-Fixes and Techno-Solutionism

**DOI:** 10.1007/s11948-024-00524-x

**Published:** 2024-12-02

**Authors:** Henrik Skaug Sætra, Evan Selinger

**Affiliations:** 1 Department of Informatics, University of Oslo, Oslo, Norway; 2 Dept. Philosophy, Rochester Institute of Technology, Rochester, USA

**Keywords:** Techno-fix, Techno-solutionism, Social problems, Conceptual engineering, Ethical evaluation

## Abstract

Can technology resolve social problems by reducing them to engineering challenges? In the 1960s, Alvin Weinberg answered yes, popularizing the term “techno-fix” in the process. The concept was immediately criticized and over time evolved into a disparaging term—a synonym for unrealistic technological proposals and their advocates. As the debate progressed, skepticism grew to include condemnation of a related term: “techno-solutionism.” Despite extensive criticism, both “techno-fix” and “techno-solutionism” remain ill-defined concepts. In this article, we provide more precise definitions and clearly distinguish between techno-fixes and techno-solutionism through conceptual engineering. By refining these concepts, we aim to advance the discussion and lay the groundwork for more productive analyses of the role of technology in solving social problems.

## Introduction

Everyone seems to agree that technology has positive uses. But controversy arises when people claim it can alleviate social problems by turning them into technical engineering issues. Alvin Weinberg famously endorsed this position in the 1960s when he popularized the concept of a “technological fix” (also known as “techno-fix”) (Weinberg, 1966).

Critics immediately characterized Weinberg’s vision as an unrealistic and often unwise shortcut (Johnston, 2020). Over the years, the criticism has persisted, and, thanks to Evgeny Morozov’s *To Save Everything Click Here* (Morozov, 2013), the conversation has evolved to include condemnation of “solutionism” (also referred to as “technological solutionism” and “techno-solutionism”). Much like detractors of technological fixes, Morozov depicted solutionism as misguided technological optimism—a fallacious way of thinking and possibly dishonest grift.

Today, scholars, civil rights activists, and journalists demonize techno-fixes and techno-solutionism (sometimes using the terms interchangeably) and their advocates. For example, Gillespie (2009) laments the naïveté of believing “we can somehow simply and justly resolve social problems with technologies.” Like many Science and Technology scholars and Philosophers of Technology, he argues that appeals to technological fixes “bypass crucial questions” about the “social bases” of any given problem (Gillespie, 2009).

Given the scope of past and present discussions of techno-fixes and techno-solutionism, it is crucial to ensure that critics use these terms clearly, consistently, and fairly. After all, they have been applied to the following types of issues: poverty and riots (Weinberg, 1966), illiteracy (Sarewitz & Nelson, 2008), education (Black, 2022; Morozov, 2022), public health (Fox Cahn, 2021; Mann et al., 2022; Maschewski & Nosthoff, 2023; Selinger, 2020), mental health (Berners-Lee, 2023), and child safety online (Angel & Boyd, 2024). Relatedly, the United Nations characterizes many of its Sustainable Development Goals as social problems that can likely be fixed by technology and growth (Sætra, 2023). It is far from the only global organization to use these terms to frame its main goals.

Unfortunately, despite extensive criticism, which recently has been extended to issues like artificial intelligence (Selinger, 2023), crypto currencies (Allen, 2024), and surveillance (Fox Cahn, 2022), “techno-fix” and “techno-solutionism” remain ill-defined. The imprecision and semantic slipperiness have created two significant problems. First, some appeals to these concepts distort technology’s potential and limitations. Second, these concepts have been used to unfairly justify condemning a techno-optimist’s character and lack of foresight. Consequently, while there are valid reasons for criticizing techno-fixes and techno-solutionism, these terms require a more precise analysis.

To this end, our paper has two goals: (1) clarify the key differences between “techno-fix” and “techno-solutionism” by redesigning the concepts, and (2) prepare the ground for applying the revised concepts in practical contexts. We proceed as follows. In Sect. 2, we clarify our method, which aligns with the main goals of conceptual engineering—the analysis, improvement, and implementation of concepts. In Sect. 3, we apply conceptual engineering to explain the differences between “techno-fixes” and “techno-solutionism,” considering natural language use, reviewing relevant literature, and addressing conceptual ambiguities. Finally, we conclude by discussing the implications of our refined concepts for future research and practice.

## Conceptual Engineering

We are taking a conceptual engineering approach to understanding techno-fixes and techno-solutionism. Although the analysis and design of concepts date as far back as the origins of philosophy, conceptual engineering structures such efforts and introduces a more systematic way to approach the *assessing*, *improving*, and *implementing of* representational devices (concepts) (Cappelen & Plunkett, 2020). Chalmers (2020) compares these activities to using knowledge to analyze, design, and build objects. These views build upon earlier discussions. For example, Richard Creath characterized the logical positivist Rudolf Carnap—who referred to his philosophy as “linguistic engineering”—as a conceptual engineer (Chalmers, 2020; Creath, 1990). Today, Herman Cappelen is one of the leading proponents of conceptual engineering, in part due to his influential book *Fixing Language: An Essay of Conceptual Engineering* (Cappelen, 2018).

Our conceptual engineering approach to “techno-fixes” and “techno-solutionism” follows the following procedure. To assess/analyze these concepts, we begin by critically examining how “fixes” and “solutions” are used in natural language across a range of representative cases. We evaluate their strengths and weaknesses by looking at four criteria: (1) alignment with natural language and intuitive appeal to experts and non-experts, (2) how clear the definitions are, (3) how easily the definition can be consistently and usefully applied to relevant examples, and (4) whether definitions are maximally normatively neutral or biased in ways that reflect controversial normative assumptions. We select the most neutral definitions for one overarching reason: They are the most pragmatically useful. Terms that elicit controversial normative assumptions are more likely to be deployed as dog whistles by limited, partisan communities than become widely accepted vernacular.

Once we have analyzed the natural language components of the concepts, and how they have historically been and is currently used, we re-engineer “techno-fix” and “techno-solutionism” to improve them according to the four criteria. For this design phase, we aim to create a vocabulary that scholars, activists, and journalists who hold diverse ethical and political beliefs can widely embrace. Finally, for the implementation component, we provide examples of the new concepts in use and clearly summarize their commonalities and differences. To this end, we prepare the ground for the future use of the concepts in line with our proposed changes to them.

## Re-building Concepts: Techno-fixes and Techno-solutionism

Thomas Nickles once asked, “What is a problem that we may solve it?” (Nickles, 1981). “Social problems” are harder to define than most people believe because the categories “social” and “problem” both pose interpretative challenges. To improve the definitions of “techno-fixes” and “techno-solutionism,” we will examine how the terms “fix” and “solution” are used in everyday language and consider their relation to “fixing” and “solving” problems.

### Fixes and Techno-fixes

Natural language suggests a potential and tentative key difference between fixes and solutions. The word “fix” tends to imply that a problem can be resolved relatively easily and quickly, as in a “quick fix” and “easy fix.” Indeed, this is how critics tend to understand the word “fix” in techno-fix proposals (Rosner, 2004). “Fixes” tend to be paired with fast and basic remedies. A “solution” better reflects attempts to address complex problems, and not necessarily quickly and easily. This framing aligns with Weinberg’s description of a techno-fix as quick and easy band-aid approach that mainly addresses symptoms of a problem. Sometimes, as Weinberg notes, band-aids are appropriate. Addressing symptoms does not necessarily mean one is ignorant of or indifferent to the underlying causes.

Furthermore, claiming that a problem can be fixed implies that we are dealing with something broken that can be mended. For example, a shelf can be fixed, and so can a wheel with a broken spoke. In both cases, an original state—or a close approximation—is restored. However, there are two reasons why, for example, declaring that poverty can be “fixed” sounds naïve. First, the problem is far more complicated than repairing a broken wheel. Second, there is no original social condition, an Edenic moment, when poverty—as we use this term today—did not exist. However, a “band-aid fix” could, for example, entail having the state write a check to all or parts of the population, to help alleviate the worst consequences of poverty. As with the bleeding child in need of a band-aid, it will not solve any underlying causes, but it *can* help address current suffering in a situation where harms have already occurred. This could perhaps be characterized as a “policy-fix” or “money-fix” as contrasted with a techno-fix.

To be sure, one can–and often does–use “fix” and “solve” interchangeably. Some might say that they have “solved” the problem of having run out of milk by going to the store, and others might want to “fix” the international state system and end all wars. Despite this, our proposed distinction has merit. We will show that the distinction is *useful* for highlighting some of the major differences between techno-fixes and techno-solutionism. It is also *reasonable* because it corresponds with the most common use of the terms. It also satisfies our goal of maintaining normative neutrality; neither fixes nor solutions are inherently bad in their natural language uses.

### *Techno*-fixes

Weinberg was an unabashed technological optimist, and purportedly “promoted the belief that technological innovation could resolve any social issue as an article of faith” (Johnston, 2018). Echoing the longstanding view that humans are *homo faber—*a species that can do things no other species can when making and using tools–and encouraged by profound techno-scientific accomplishments, he saw great potential in science and technology. Such optimism is easy to understand. He lived at a time of large-scale technological breakthroughs, including the moon landing (which remains so deeply embedded in the innovator’s psyche that we still characterize tough challenges as moonshots) and the harnessing of nuclear power for constructive and destructive purposes.

As an example of a seemingly perennial social problem, let us briefly consider the second Sustainable Development Goal: *Zero Hunger*. It specifies the goals of eliminating hunger by 2030, achieving food security, improving nutrition, and promoting sustainable agriculture (United Nations, 2015). Now, if some people are starving while others have a lot of food, the situation can be understood as a social problem that is rooted in the unjust distribution of goods and power. This social framing further suggests social problems are deeply interconnected. For example, SDG 2 relates tightly to other goals, such as SDG 1 (No Poverty) and SDG 10 (Reduced Inequalities). However, the issue of hunger can be reframed in a way that prioritizes a technological response. What if someone invented a new technology or better implemented an existing one that could dramatically increase food production and effectively eliminate hunger? According to techno-fix advocates like Weinberg, this would be a technological fix for a social problem. While you might prefer a social or political framing, the technological framing should not be considered *wrong*, if it is plausible, or at least possible. The history of human society does in fact lend some credence to the idea that science and technology has and can dramatically change and from certain perspectives improve food systems. Not without costs, of course, but as we will argue below, all proposed fixes–including non-technological–have costs.

In the same spirit as the hypothetical agricultural technology optimists, Weinberg asked us to consider how the invention of birth control technologies like intrauterine devices lessened social problems related to gender inequality and discrimination. He also proposed we could “fix” problems related to riots and social unrest by providing Black people with air conditioners and free electricity. And, as the continuation of an earlier and popular notion (famously proclaimed by Robert Oppenheimer), he characterized nuclear weapons as a technological fix to war that would bring about lasting peace (Weinberg, 1966). There is no need to explore these examples in detail. Some techno-fix proposals have been outrageously foolish for obvious social, political, and cultural reasons, while others have been highly controversial and contested. Still, we should not summarily dismiss them. Nuclear deterrence and the idea that nuclear weapons might prevent large scale wars, for example, has long been and is still relatively mainstream in the field of international relations (Narang, 2014).

Additional pedestrian examples of techno-fixes have also been proposed. Howard Scott, the founder and director of the Technocracy Inc. movement and predecessor of the line of thinking that Weinberg continued as he introduced the term techno-fixes, often used the example of streetcar design when explaining how technology can solve problems (Johnston, 2020). Open streetcars allowed people to jump on—and fall off—and this affordance created safety hazards that could be seen as a social problem. Heralding the power of engineering, Scott contrasted ineffective *bans* against riding on the platforms or fines for doing the same to the *technological* solution of designing closed streetcars. Instead of turning to law or economic incentives to motivate behavioral change, he argued that technology could effectively bypass the problem (Johnston, 2020). This example is often seen as an archetypical techno-fix – one that conveniently brackets the issue of engineers putatively poorly designing streetcars in the first place.

Weinberg believed that a key benefit of the techno-fixes was that it allowed us to address social problems without requiring people to significantly modify their behavior and without needing to forego short-term benefits to meet long-term ones (Weinberg, 1966). He thus characterizes technological fixes as superior to “social engineering” proposals that require transformational behavioral change (Weinberg, 1966).

At its core, Weinberg’s approach is based on profound skepticism—doubt that individuals can be rationally convinced to change their established ways to make better choices. Oelschlaeger (1979) contends that techno-fix proponents presuppose that humans—specifically those living in advanced industrial societies—tend to prioritize their short-term well-being and overly discount future outcomes. More vividly stated, Oelschlaeger claims that techno-fix adherents presuppose modern Western humans are overly-consumptive, pleasure-maximizing “greedy-little-pig” people. In this spirit, Weinberg also used expressions like “natural shortcomings” when describing his sense of our profound limitations.

This sounds gloomy, but techno-fix proponents counterbalance their skepticism about individuals typically being amenable to and capable of freely making certain types of change with optimism about our species ingenuity (Oelschlaeger, 1979). Weinberg insisted that “The Technological Fix accepts man’s intrinsic (or natural) short-comings and circumvents them or capitalizes on them for socially useful ends. Through collective science and technology efforts, human reason can be harnessed to fix the problems human nature tends to create. The Technological Fix is, therefore, eminently practical and, in the short term relatively effective” (Weinberg, 1966). In other words, “One does not wait around trying to change people’s minds.” (Weinberg, 1966).

However, the use of the concept techno-fix has changed radically since Weinberg’s time. In Rosner’s (2004) exploration of techno-fixes, she notes that critics mainly use the term to refer to the fallacy of “using inappropriate technology” for “a quick, cheap fix”; doing so, they insist, creates “more problems” than it solves. Relatedly, Huesemann and Huesemann (2011) link techno-fixes to excessive techno-optimism. Beginning from the premise that technology inevitably yields negative unintended outcomes, they conclude that techno-fixes can only provide temporary, but never sustained, relief.

By contrast, we believe it is important to be more open-minded when defining “techno-fix” and not judge, a priori, that they all inevitably will fail. This is in line with, for example, Scott (2011) and Nydal et al. (2023), who argue that the techno-fix should not be defined as a fallacy and used exclusively as a dismissive term. To completely foreclose even the possibility of success—however unlikely it may be—is to be guilty of three mistakes. First, the position conflates inductive reasoning with deductive reasoning. In other words, it extrapolates from past empirical failures to future ones in a non-probabilistic manner. Second, the view is incompatible with pluralism regarding both legitimate disagreements about the likely consequences of a technological intervention *and* the fact that people might evaluate the same sets of consequences differently based on them holding a set of different and incommensurable values. Third, it tends to ignore real challenges related to changing human behavior rapidly and downplay the positive potential of new technological breakthroughs.

In other words, the ardent techno-fix critic might lack the moral imagination to conceive of a possible community that would reasonably judge a techno-fix as likely *enough* to be successful based on its guiding principles and values to prefer it to other interventions. By changing the concept to something inherently critical, the concept has become more of a dog whistle for signaling a certain perspective and attitude to socio-technical analysis than a normatively neutral concept describing certain forms of technological interventions – a change we consider to be unfortunate.

### Techno-fixes Defined

Here, then, is our proposed definition of “techno-fix”, which will later be contrasted with techno-solutionism:

- A *techno-fix* is a technological remedy for a social problem that only involves minimal use of political power or economic incentives to change an individual’s behavior and does not require changing social norms.

This definition aligns well with our criteria for re-engineering: it pairs well with natural language, retains normative neutrality, and corresponds with most of the literature on techno-fixes (from its origin to more recent uses). The main change is removing the normative evaluation, if not stigma, that has become pervasive. Based on this definition, here are illustrative initiatives that aligns with our proposed definition.

- Carbon-capture and storage solutions aimed at reducing GHG emissions without society significantly changing economic and energy systems.
- Electric vehicles reducing air pollution and GHG emissions without society significantly changing transportation routines or radically altering city infrastructure from car-based to alternative transportation systems.
- Nuclear power producing enough carbon-free energy to maintain energy-intensive industries.
- Assistive robotics enabling current eldercare systems to persist with changing demographics and a shortage of human healthcare workers.
- The increasing use of drugs intended initially to treat diabetes to combat obesity without people significantly changing their eating and exercise habits or society significantly changing its food systems.

We recognize, of course, that there are many well-known objections to these endeavors. For present purposes, they merely serve as preliminary examples of how technology potentially can be used to solve social problems while imposing minimal change in individual behavior and pervasive norms.

### Solutions and Techno-solutionism

“Technological solutionism” is a more recently developed concept than “techno-fixes.” It builds on the idea of “solutionism,” a term that has been used intermittently since the 1950s. However, to improve the concept techno-solutionism, we also here argue for a return to natural language to highlight the contrast between a fix and a solution.

A solution could be understood as “a means of… dealing with a difficult situation” (Solution, 2018). A band-aid is hardly a *solution* in this sense. Furthermore, a solution does not necessarily address a typical and clearly identified “problem.” It could, for example, detail how to respond to changing circumstances or how to best utilize the potential of some new technology. While all such challenges can be framed as problems, they are not *typical* problems. Consequently, while “fixes” tend to be paired with fast and basic remedies, a “solution” better reflects attempts to address complex problems, and not necessarily quickly and easily.

We saw above that it might be naïve to seek a “fix” for complex social problems. However, it often makes more sense to talk about finding *solutions* to, for example, poverty—changing aspects of society to bring about something new and better. This *could* be fueled by romanticism and based on naïve attempts to bring “back” some idealized conceptualization of historical states of nature; it also could and often will be based on more ambitious attempts to bring about something better than what we have had.

Johnston (2020) refers to three historical uses of “solutionism.” The oldest is Samuel P. Huntington’s, 1957 evocation in *The Soldier and the State* (Huntington, 1957). Huntington appealed to “solutionism” as a contrasting ideal to “conservatism.” He wrote: “Present in virtually all the strands of the new conservatism were a stress on the limitations of man, an acceptance of institutions as they were, a critique of utopianism and ‘solutionism,’ and a new respect for history and society as against progress and the individual.” Conservatism, thus portrayed, is clearly not necessarily something to strive for. Perhaps a healthy dose of solutionism is *required* to change our societies for the better?

Then, there is Edward Hodnet’s (1959) book *The Art of Working With People*. Hodnet warned against “solutionism—the flabby optimism that there is a simple answer and that it will yield to the magic of a personality, brainstorming, sitting down and talking things over, or other tribal nostrums.” Johnston’s final example is Daniel Fox’s (1963) book *Engines of Culture*. Fox depicts “technocratic solutionism” as a rejection of politics. He argues that those “who practice solutionism insist that problems have technical solutions even if they are the result of conflicts about ideas, values and interests.”

However, Johnston does not present a complete review of the origins and uses of solutionism. More recent uses include D. P. Baker’s, 1984 description of solutionism as a “hankering” or “passion” (Baker, 1984). Baker claims “[s]olutionism is just a belief that for every problem there exists a solution; and successful persons are those who solve problems” (Baker, 1984). Then, in 2005, Gilles Paquet makes the following assertion in *The New Geo-Governance: A Baroque Approach* (Paquet, 2005): “The ‘liberal constitutional project’…is predicated on the belief that only the central government has the capacity to appreciate the nature of today’s problems, and to suggest meaningful solutions. ‘Solutionism’ or ‘ultrasolutionism’ is indeed the name of the game: issues are interpreted as puzzles to which there is a solution, rather than problems to which there may be a response” (Paquet, 2005). This latter definition captures a key potential difference between techno-fixes and techno-solutionism, that we will build upon below.

### Techno-solutionism

More recently, we have Evgeny Morozov’s, 2013 *To Save Everything*,* Click Here: The Folly of Technological Solutionism* (Morozov, 2013), which uses “solutionism” and “techno-solutionism” synonymously. Most current analyses and applications of these concepts now explicitly refer to Morozov (Black, 2022; Selinger, 2023). However, some analysts, like Kuusela and Kantola (2023), make *some* effort to trace or interrogate the concept, referring to how Michael Dobbins invented the idea in his 2009 book *Urban Design and People* (Dobbins, 2009), whereas Morozov subsequently made it “famous.” As we have seen, however, the term goes back much further.

In parallel with the criticisms of techno-fixes, Morozov treats solutionism as a fallacy, and others have agreed with him (Selinger, 2023). The concept now often bakes in the normative evaluation of the actors and interventions involved, making it a highly normatively charged concept. Morozov adapted the term from architecture and urban planning and sees it as an “unabashedly pejorative term” that refers to “sexy, monumental, and narrow-minded solutions… to problems that are extremely complex, fluid, and contentious.” Whereas Weinberg emphasized government techno-fixes, Morozov focuses on the private sector and portrays solutionism as an aspirational mindset that has gained prominence in the digital age thanks to Silicon Valley entrepreneurs. On the one hand, he maintains that solutionism has all the flaws of techno-fixes. On the other hand, Morozov contends that solutionism is a different ideal because of the distinctive manner by which its adherents incorrectly frame social problems.

Rather than acknowledging the inherent complexity of social life, Morozov insists that today’s solutionists myopically focus on limits that the affordances of digital technology are well suited to address: eliminating inefficiencies, reducing ambiguities, and decreasing opacity. For example, in the early years of social media, platforms like Facebook were presented as a solution to the problem of global misunderstanding and tribalism. The guiding principle was that people would get along better if they could more easily communicate with one another to dispel unfair preconceptions, like prejudices. However, as Morozov sees it, this emphasis on eliminating friction that slows communication is the wrong way to frame the problem. It naively oversimplifies the complex social, political, and economic reasons that long-standing animosities and suspicions exist. From Morozov’s perspective, solutionists frequently invent fake problems. However, it seems clear that others might disagree with Morozov both on the evaluation of the likely consequences of the use of technology *and* the desirability of the situation it might lead to.

According to Morozov sometimes solutionists oversimply and overlook important factors because they have, in good faith, adopted an extreme engineering mindset. And sometimes, Morozov alleges, solutionists have nefarious motives. While promoting a product or company as the solution, they rhetorically disguise their self-serving interests as altruism. However, we can rarely be confident about others’ motives and intentions. Furthermore, the idea that self-interested actors in a relatively free market situation generates social benefits is certainly not an impossibility, even if one might disagree about what should be the relative strength of state and markets. Packing all sorts of evaluations related to not just the use of technology, but also intentions, political, and economic ideology, along with evaluations of the same into the concept, makes this version of the “techno-solutionism” concept bloated and inaccessible to the uninitiated reader.

Morozov has more recently characterized solutionism in political-economic terms as “digital neoliberalism.” He defines it as the reframing of “social problems in light of for-profit technological solutions” that favor privatization and the marketization of public concerns (Morozov, 2023). This account suggests solutionism is fundamentally a political problem tightly related to economic and political economy. Solutionists, in Morozov’s eyes, cannot envision feasible responses to social problems that require rethinking and even contesting the power imbalances of market forces and the government’s abdication of its responsibility to promote public welfare. However, we have already seen that solutionism as the term has been used historically entails exactly this radical approach that Morozov seems to deny, one that is contrasted with conservatism.

Two questions arise in light of these uses of the concept. How, exactly, can “solutionism” be distinguished from “techno-solutionism”? And are there compelling reasons for presenting a more open-minded definition of “techno-solutionism” that does not categorically denounce it as a fallacy? In other words, can we justify viewing techno-solutionism in a slightly more favorable light, much like we did “techno-fixes” in the previous section? The criteria of normative neutrality, for example, provides such a reason.

To answer these questions, we need to address four preliminary issues. First, while Morozov uses the terms “solutionism” and “techno-solutionism” interchangeably, it is more accurate to characterize techno-solutionism as a subtype of solutionism. This helps with the criteria we established related to increased conceptual clarity. Specifically, techno-solutionism is a technologically oriented means of trying to achieve solutionist goals. Other options for pursuing solutionism include but are not limited to, attempts to reorganize societies or organizations through political, legal, social, and economic initiatives. Indeed, there is explicit language in proposals for “economic solutionism” (Westerfield, 1963) and “legislative solutionism” (Sarachild, 1978).

Second, defining solutionism as a distinctive outlook requires demarcating it from alternatives—including the vision of addressing social problems that underlies techno-fix advocacy. Because “solutionism” is used in many different ways, there is no perfect way to do this that covers every case. For example, Hodnet’s (1959) focus on “simple answers” matches how many people see one of the most important things about techno-fixes. Furthermore, some of Morozov’s examples of solutionism—particularly in his early criticisms of oversimplistic apps—may just as easily be classified as techno-fixes. Indeed, the title of STS scholar Jack Stilgoe’s reflection on *To Save Everything Click Here* is “The Techno-Fix is In” (Stilgoe, 2023). It begins by reminding us of Weinberg’s agenda and builds up to the following assertion: “According to Evgeny Morozov, the technological fix is alive and well.” Given these constraints, there are two main ways to deal with techno-solutionism. One is to agree with Stilgoe (2023) and Johnston (2020) that it is mainly a different word for techno-fixes. The other is to elucidate the differences brought up above—and expressed by Paquet—which provides us with the means to distinguish between different types of technological interventions. We have chosen the latter strategy for the reasons provided in the introduction.

A reasonable way to differentiate solutionist interventions from techno-fixes is to see them as more likely to accept significant societal changes and changes to social norms and behaviors. This difference is easiest to see when considering examples of techno-solutionism. When Zuckerberg proposed to unite the world through Facebook, his solution was for a disruptive technology to fundamentally alter how people communicate. His idealization of how the “metaverse” will normalize the persistent use of augmented and virtual reality focuses on similar themes (Selinger, 2022). There are many other disruptive techno-solutionist proposals. They include ambitious attempts at terraforming, large-scale deployment of surveillance and information systems in all areas of society to optimize political and social systems, and using biotechnology to improve and change humans as a species (e.g., transhumanism).

These types of technical applications, which favor notably different and, presumably, better ways of living, differ from quick and simple uses of technological band-aids to fix social problems. Another example is the recent surge in proposals concerning the revolutionary potential of artificial intelligence. Some, like Mustafa Suleyman, CEO of Inflection AI and former co-founder of Google DeepMind, believe that “[p]ersonal AI is going to be the most transformational tool of our lifetimes” (Wiggers, 2023). Technologist and venture capitalist Marc Andreesen also articulated high hopes. He received a lot of attention for his proclamation that AI “will save the world” and potentially provide a “way to way to make everything we care about better” (Andreessen, 2023).

Since solutionist ideas tend to be radical or at least call for overt social change, it’s harder to deny that they have a political dimension than techno-fix suggestions. Solutionists can, of course, reject the accusation of politics and insist that their stance is ideologically neutral in some way. However, since their proposals typically lead to debates about important tradeoffs, it is a hard case to make.

Third, since Morozov takes a polemical approach to discussing solutionism, his analyses tend to be written in a way that leaves the reader suspicious of the solutionist’s underlying motivation – demonstrating how techno-solutionism, much like techno-fix, has become somewhat of a suggestive dog whistle. We think that he might be correct about the people promoting technology in certain cases—and that there are good reasons to be wary of rampant bad faith. However, combining the principle of charity (a principle of discourse ethics that obligates us to try to interpret other people’s claims fairly and respectfully) with respect for value pluralism requires us to differentiate the definition of solutionism from valid criticisms of it.

In other words, tech entrepreneurs may deceive the public with platitudes about making the world a better place. Or they may sincerely make these claims. Without explicit statements of a solutionist’s underlying thoughts—like a whistleblower releasing a recording where their insidious intentions are revealed—it can be challenging to determine the real motivation. Like everyone else, people in the tech industry can have complex inner lives and act for mixed reasons. Indeed, a solutionist critic’s perspective may be so different from that of a tech entrepreneur that the critic misses the big picture. In cases where a critic is displeased by an entrepreneur’s self-interested aims, it is worth keeping one thing in mind: egocentric people can create socially beneficial change.

Fourth, Morozov’s contention that the solutionist invents problems raises the same interpretive concerns as his take on their motivations. While this may be a common fault, accepting pluralism necessitates being open to the following possibility. What appears to be an incorrect diagnosis of the fundamental cause of a social problem may be a creative proposal that is difficult to grasp because it profoundly challenges current thinking.

Finally, we note that the literature on solutionism and techno-solutionism does not explicitly state a desire to overcome or avoid challenges associated with human nature. In fact, solutionism is best characterized as a type of *social engineering*, making the contrast to techno-fixes quite clear. When Karl Popper emphasized piecemeal social engineering, he meant incremental social engineering procedures that aid in the “transformation of society” toward “social progress” and “new developments and problem solutions” (Stelzer, 2016). Popper’s analysis was founded on his ardent belief that logic and science may pave the way to the aim of building an “open society.” Although Popper was dubious of large-scale and revolutionary societal change, solutionist optimism in the possibility of drastic and quick transformation simply shows a different point of view on which type of solutionism is preferable.

That said, solutionism does not have to be a commitment to rapid, transformative change. On the contrary, the goal can be to make a meaningful contribution to changing society over time through new and innovative pathways. Indeed, solutionist interventions can be gradual and require many steps over long periods.

### Solutionism and Techno-solutionism Defined

Given the above considerations, here are our definitions:

- Solutionism is a way of thinking about social problems. It is characterized by confidence in human reason and our capacity to find and implement numerous strategies for overcoming obstacles and capitalizing on opportunities, typically through courses of action that significantly alter social norms.
- Techno-solutionism is a technology-driven approach to solutionism. It is rooted in Promethean optimism about the revolutionary potential of science and engineering.

This way of putting things increases conceptual clarity in two ways. They eliminate some of the baggage that has been added in recent years, while also clearly distinguishing between solutionism and techno-solutionism. Since the new concept of “techno-solutionism” is normatively neutral, critics still can use it characterize a certain type of technological intervention or attitude. However, their reasons for denouncing advocates will require additional analysis; it is not justified by using the concept alone. We also remain much closer to the core content of the natural language use of “solutions,” while making the concept more intuitively accessible for non-specialists.

Here are examples of technological interventions that illustrate our redesigned concept:

- More radical forms of geo-engineering aimed at actively managing Earth systems and the climate.
- Proposals for replacing human institutions and decision-making processes with computational systems and data for more effectively managing, for example, a country’s economy and policies more generally. Such longings for technocratic solutionism have existed long before modern AI systems – Chile’s *Cybersyn* is one example – but recent technological developments and increased data availability makes such solutions more alluring and seemingly realistic.
- Smart cities and societies initiatives aimed at datafying and managing entire communities – both social relations and sets of infrastructures – to improve services, lives, but also to generate genuinely new opportunities and new forms of communities.

### Fundamental Commonalities and Differences

So far, we have claimed the following questions can help distinguish “techno-fixes” from “techno-solutionism.” Is technology being used to address social problems without requiring people to make significant behavioral changes? Alternatively, is it to help us develop and construct new and better societies—ones where common social norms and habits are acceptable targets of technological intervention? The concept of a “fix” suggests the former, while “solutions” correspond with the latter. This is a useful baseline distinction.

On the surface, both concepts share a foundational optimism about technology’s potential to alleviate social problems. They reflect optimism that human ingenuity, manifested through scientific and technological innovation, can overcome significant societal challenges. Nevertheless, they are also clearly distinct. Table 1 summarizes our main insights.

**Table 1:** Table of key aspects and differences

	Techno-fixes	Techno-solutionism
*Technological Approach*	Provides targeted, technical remedies to specific problems	Uses technology for broad societal transformation
*Scope of Change*	Minimal disruption; maintains existing social norms	Significant change; alters social norms and behaviors
*Time frame*	Short-term focus	Short-term and long-term focus
*Political Orientation*	Conservative; preserves status quo	Radical; advocates for systemic change
*View of Human Nature*	Accepts existing behaviors; bypasses need for change	Seeks to modify human behavior and culture
*Role of Social Engineering*	Avoids extensive social engineering	Embraces social engineering through technology
*Social resilience*	May erode resilience by ignoring root causes	Aims to strengthen resilience by restructuring society
*Examples*	Carbon capture technology; Electric vehicles	Geoengineering; AI-driven societal management

### Synthesis and Implications

Our exploration of techno-fixes and techno-solutionism reveals that while both concepts share a foundational optimism about technology’s potential to alleviate social problems, they diverge in methods, objectives, and underlying philosophies. By redefining these terms with normative neutrality, we have aimed to eliminate semantic confusion and provide clearer distinctions that can facilitate more productive discussions.

Understanding these distinctions is crucial for several reasons. First, it allows for more precise communication among scholars, policymakers, and practitioners when discussing technological interventions. Clear definitions help prevent the conflation of different types of technological optimism and avoid unfairly maligning advocates based on ambiguous terminology.

Second, our refined concepts highlight the importance of considering the broader societal implications of technological remedies. Recognizing whether a proposed intervention aligns more closely with a techno-fix or techno-solutionist approach can inform assessments of its potential impact on social norms, political structures, and individual behaviors.

Finally, acknowledging the fundamental differences between techno-fixes and techno-solutionism underscores the significance of value pluralism in debates about technology’s role in society. Disagreements over technological interventions often stem from differing value systems and visions of the good life. By appreciating this plurality, we can foster more inclusive and constructive discussions that respect diverse perspectives.

## Conclusion: Promoting Ethically Conscientious Discussion

In this article, we have critically examined the terms “techno-fix” and “techno-solutionism,” addressing the conceptual confusion that has hindered productive discussions about technological interventions in social problems. Through the process of conceptual engineering, we have proposed refined definitions that distinguish these two terms more clearly. Our analysis facilitates clearer discussions and can aid in future ethical evaluations conducted by others.

A key takeaway from this analysis is the importance of embracing value pluralism when assessing the role of technology in addressing social problems. Rather than dismissing techno-fixes and techno-solutionism outright as misguided or fallacious, we argue for a more nuanced approach that considers the diverse perspectives and values that inform people’s attitudes toward technological optimism. Recognizing the potential of both approaches while remaining vigilant about their limitations allows for more balanced and responsible technology governance.

The refined conceptual framework developed here has broad implications. It can help stakeholders critically assess whether specific technological interventions are appropriate for the problems they aim to address and whether they are aligned with the ethical principles of inclusivity, sustainability, and societal well-being. Ultimately, by fostering clearer distinctions and promoting ethical analysis, we aim to advance a more productive and conscientious discussion about the role of technology in solving social problems. This approach not only enriches academic debate but also guides practical decision-making, contributing to more thoughtful and effective applications of technology in society.

## References

[CR1] Allen, H. J. (2024). Fintech and techno-solutionism. Available at SSRN. 10.2139/ssrn.4686469

[CR2] Andreessen, M. (2023). *Why AI will save the world*. Andreessen Horowitz. Retrieved July 10 from https://a16z.com/2023/06/06/ai-will-save-the-world/

[CR3] Angel, M. P., & boyd (2024). *Techno-legal solutionism: Regulating children’s online safety in the United States*. https://www.danah.org/papers/2024/Techno-legal_Solutionism_PREPRINT.pdf

[CR4] Baker, D. P. (1984). *The library media program and the school*. Libraries Unlimited.

[CR5] Berners-Lee, B. (2023). Reconciling healthism and techno‐solutionism: An observational study of a digital mental health trial. *Sociology of Health & Illness*. 10.1111/1467-9566.13683 10.1111/1467-9566.13683 37337395

[CR6] Black, S. (2022). Marx’s ghost in the shell: Troubling techno-solutionism in post-secondary education and training policy imaginaries. *Education as Change*, *26*(1), 1–23. 10.25159/1947-9417/11188

[CR7] Cappelen, H. (2018). *Fixing language: An essay on conceptual engineering*. Oxford University Press.

[CR8] Cappelen, H., & Plunkett, D. (2020). Introduction: A guided tour of conceptual engineering and conceptual ethics. In A. Burgess, H. Cappelen, & D. Plunkett (Eds.), *Conceptual engineering and conceptual ethics*. Oxford University Press.

[CR10] Creath, R. (Ed.). (1990). *Dear Carnap, Dear Van: The Quine-Carnap correspondence and related work*. University of California Press.

[CR11] Dobbins, M. (2009). *Urban design and people*. John Wiley & Sons, inc.

[CR14] Fox, D. M. (1963). *Engines of culture: Philanthropy and art museums*. Transaction.

[CR12] Fox Cahn, A. (2021). I forged New York’s digital vaccine passport in 11 minutes flat. *Daily Beast*. https://www.thedailybeast.com/i-forged-new-yorks-digital-vaccine-passport-in-11-minutes-flat

[CR13] Fox Cahn, A. (2022). The ‘surveillance solutionism’ of putting cameras in NYC subways. *Wired*. https://www.wired.com/story/the-surveillance-solutionism-of-putting-cameras-in-nyc-subways/

[CR15] Gillespie, T. (2009). Wired shut, copyright and the shape of digital culture. *Journal of Information Communication and Ethics in Society*, *7*(2/3), 213–218.

[CR16] Hodnet, E. (1959). *The art of working with people*. Harper.

[CR17] Huesemann, M., & Huesemann, J. (2011). *Techno-fix: Why technology won’t save us or the environment*. New Society.

[CR18] Huntington, S. P. (1957). *The soldier and the state: The theory and politics of civil–military relations*. Belknap.

[CR9] Chalmers, D. J. (2020). What is conceptual engineering and what should it be? *Inquiry*, 1–18. 10.1080/0020174X.2020.1817141

[CR19] Johnston, S. F. (2018). The technological fix as social cure-all: Origins and implications. *IEEE Technology and Society Magazine*, *37*(1), 47–54. 10.1109/MTS.2018.2795118

[CR20] Johnston, S. F. (2020). *Techno-fixers: Origins and implications of technological faith*. McGill-Queen’s University.

[CR21] Kuusela, H., & Kantola, A. (2023). Unpolitical solutionism: Wealth elite sentiments against democracy and politics. *The British Journal of Sociology*. 10.1111/1468-4446.13043 10.1111/1468-4446.13043 37370212

[CR22] Mann, M., Mitchell, P., & Foth, M. (2022). Between surveillance and technological solutionism: A critique of privacy-preserving apps for COVID-19 contact-tracing. *New Media & Society*, *14614448221109800*. 10.1177/14614448221109800

[CR23] Maschewski, F., & Nosthoff, A. V. (2023). Pandemic solutionism. *Digital Culture & Society (DCS),**8*(1/2022), 14–-66.

[CR24] Morozov, E. (2013). *To save everything, click here: The folly of technological solutionism*. Public Affairs.

[CR25] Morozov, E. (2022). *Avoiding solutionism in the digital transformation of education*. UNESCO Futures of Education Ideas LAB. Retrieved June 23 from https://en.unesco.org/futuresofeducation/ideas-lab/morozov-avoid-solutionism-digital-transformation

[CR26] Morozov, E. (2023). The true threat of aritificial intelligence. *New York Times*. https://www.nytimes.com/2023/06/30/opinion/artificial-intelligence-danger.html

[CR27] Narang, V. (2014). *Nuclear strategy in the modern era: Regional powers and international conflict*. Princeton University Press.

[CR28] Nickles, T. (1981). What is a problem that we may solve it? *Synthese*, 85–118.

[CR29] Nydal, R., De Grandis, G., & Ursin, L. (2023). When is a techno-fix legitimate? The case of viticultural climate resilience. *Journal of Agricultural and Environmental Ethics*, *36*(1), 3. 10.1007/s10806-023-09900-2

[CR30] Oelschlaeger, M. (1979). The myth of the technological fix. *The Southwestern Journal of Philosophy*, *10*(1), 43–53.

[CR31] Paquet, G. (2005). *The new geo-governance: A baroque approach*. University of Ottawa.

[CR32] Rosner, L. (2004). Introduction. In L. Rosner (Ed.), *The technological fix: How people use technology to create and solve problems*. Routledge.

[CR33] Sætra, H. S. (2023). The role of technology in alternatives to growth-based sustainable development. In H. S. Sætra (Ed.), *Technology and sustainable development: the promise and pitfalls of techno-solutionism* (pp. 249–264). Routledge. 10.1201/9781003325086-18

[CR34] Sarachild, K. (1978). *Feminist revolution*. Random House.

[CR35] Sarewitz, D., & Nelson, R. (2008). Three rules for technological fixes. *Nature*, *456*(7224), 871–872. 19092910 10.1038/456871a

[CR36] Scott, D. (2011). The technological fix criticisms and the agricultural biotechnology debate. *Journal of Agricultural and Environmental Ethics*, *24*, 207–226. 10.1007/s10806-010-9253-7

[CR37] Selinger, E. (2020). 2023). The public is being misled by pandemic technology that won’t keep them safe. *OneZero*. https://onezero.medium.com/the-public-is-being-misled-by-pandemic-technology-that-wont-keep-them-safe-1966ed740a87

[CR38] Selinger, E. (2022). Metaverse myopia. *Los Angeles Review of Books*. https://lareviewofbooks.org/article/metaverse-myopia/

[CR39] Selinger, E. (2023). The delusion at the center of the A.I. boom: Rampant solutionism. *Slate*. https://slate.com/technology/2023/03/chatgpt-artificial-intelligence-solutionism-hype.html

[CR40] Solution (2018). In. *New Oxford American Dictionary*.

[CR41] Stelzer, H. (2016). Principles and policies: What can we learn from Popper’s piecemeal social engineering for ideal and nonideal theory? *Philosophy of the Social Sciences*, *46*(4), 375–391. 10.1177/0048393116639226

[CR42] Stilgoe, J. (2023). The technofix is in. *The Guardian*. https://www.theguardian.com/science/political-science/2013/mar/21/science-policy

[CR43] United Nations. (2015). Transforming our world: The 2030 agenda for sustainable development. *Division for Sustainable Development Goals*. New York, NY, USA.

[CR44] Weinberg, A. M. (1966). Can technology replace social engineering? *Bulletin of the Atomic Scientists*, *22*(10), 4–8.

[CR45] Westerfield, B. (1963). *The instruments of America’s foreign policy*. Thomas Y. Crowell Company.

[CR46] Wiggers, K. (2023). Inflection lands $1.3B investment to build more ‘personal’ AI. *TechCrunch*. https://techcrunch-com.cdn.ampproject.org/c/s/techcrunch.com/2023/06/29/inflection-ai-lands-1-3b-investment-to-build-more-personal-ai/amp/

